# Immunogenicity of panitumumab in combination chemotherapy clinical trials

**DOI:** 10.1186/1472-6904-11-17

**Published:** 2011-11-09

**Authors:** Dohan Weeraratne, Alin Chen, Jason J Pennucci, Chi-Yuan Wu, Kathy Zhang, Jacqueline Wright, Juan José Pérez-Ruixo, Bing-Bing Yang, Arunan Kaliyaperumal, Shalini Gupta, Steven J Swanson, Narendra Chirmule, Marta Starcevic

**Affiliations:** 1 Amgen Inc., One Amgen Center Drive, Thousand Oaks, CA 91320, USA

## Abstract

**Background:**

Panitumumab is a fully human antibody against the epidermal growth factor receptor that is indicated for the treatment of metastatic colorectal cancer (mCRC) after disease progression on standard chemotherapy. The purpose of this analysis was to examine the immunogenicity of panitumumab and to evaluate the effect of anti-panitumumab antibodies on pharmacokinetic and safety profiles in patients with mCRC receiving panitumumab in combination with oxaliplatin- or irinotecan-based chemotherapies.

**Methods:**

Three validated assays (two screening immunoassays and a neutralizing antibody bioassay) were used to detect the presence of anti-panitumumab antibodies in serum samples collected from patients enrolled in four panitumumab combination chemotherapy clinical trials. The impact of anti-panitumumab antibodies on pharmacokinetic and safety profiles was analyzed using population pharmacokinetic analysis and descriptive statistics, respectively.

**Results:**

Of 1124 patients treated with panitumumab in combination with oxaliplatin- or irinotecan-based chemotherapy with postbaseline samples available for testing, 20 (1.8%) patients developed binding antibodies and 2 (0.2%) developed neutralizing antibodies. The incidence of anti-panitumumab antibodies was similar in patients with tumors expressing wild-type or mutant *KRAS *and in patients receiving oxaliplatin- or irinotecan-based chemotherapies. No evidence of an altered pharmacokinetic or safety profile was found in patients who tested positive for anti-panitumumab antibodies.

**Conclusions:**

The immunogenicity of panitumumab in the combination chemotherapy setting was infrequent and similar to the immunogenicity observed in the monotherapy setting. Panitumumab immunogenicity did not appear to alter pharmacokinetic or safety profiles. This low rate of immunogenicity may be attributed to the fully human nature of panitumumab.

**Trial registration:**

ClinicalTrials.gov: NCT00339183 (study 20050181), NCT00411450 (study 20060277), NCT00332163 (study 20050184), and NCT00364013 (study 20050203).

## Background

Panitumumab is a high affinity (K_d _= 5 × 10^11 ^M) fully human IgG2 monoclonal antibody (mAb) directed against human epidermal growth factor receptor (EGFR). Panitumumab is indicated as monotherapy for the treatment of metastatic colorectal cancer (mCRC) after disease progression on fluoropyrimidine, oxaliplatin, and irinotecan chemotherapy regimens in the United States (US) and European Union (EU) [1,2]. In the US, treatment of patients whose tumors have *KRAS *mutations in codon 12 or 13 is not recommended [1]. In the EU, panitumumab is indicated for patients whose tumors express EGFR and wild-type *KRAS *[2]. Panitumumab has been shown to significantly improve progression-free survival as first-line therapy with FOLFOX4 [3] and as second-line therapy with FOLFIRI [4] in patients with mCRC tumors expressing wild-type *KRAS*.

An important concern with the administration of therapeutic proteins is the potential to induce an immune response. Immune responses against biologics can affect their pharmacokinetics (eg, alter serum concentrations), safety (by eliciting injection-site reactions or hypersensitivity), or reduce efficacy [5]. Therefore, one of the considerations for mAb therapeutic development has been to reduce the risk of undesirable immunogenicity [6]. Based on the premise that humanized or fully human mAbs would be less likely to induce an immune response than chimeric or murine-derived mAbs, engineering technologies have focused on decreasing or eliminating the presence of nonhuman sequences within the molecule. The comparison of immunogenicity rates between mAb therapeutics is challenging because of differences in dosing regimens, patient populations, and methods used to detect anti-drug antibodies. Nevertheless, it appears that the reduction in mouse sequence content has generally resulted in improved immunogenicity profiles [7], with only a few examples of fully human mAbs with high incidences of anti-drug antibody development [8,9]. Despite these advances, the immunogenic potential of a molecule is difficult to predict based on the protein sequence alone. Various additional factors may contribute to the overall immunogenicity risk, including other product characteristics (impurity profile, formulation, post-translational modifications), patient characteristics (eg, pre-existing immunodeficiency, concurrent illness), and drug administration characteristics (frequency, route, and duration) [5].

Cetuximab, an anti-EGFR chimeric mouse-human monoclonal antibody, had a reportedly low incidence of anti-chimeric antibodies as measured by a radiometric assay in early phase clinical trials [10,11]. However, a high incidence of hypersensitivity reactions consistent with IgE-mediated anaphylaxis has been observed in patients treated for mCRC in some areas of the US [12]. These hypersensitivity reactions appeared to be caused by pre-existing IgE antibodies to galactose-α-1,3-galactose, an oligosaccharide component added during the production of cetuximab in a mouse cell line by a murine-specific enzyme [13]. As expected from the apparent absence of this post-translational modification on panitumumab, hypersensitivity reactions resembling anaphylactic reactions to galactose-α-1,3-galactose have not been seen in clinical trials or postmarketing reports of patients receiving panitumumab. Additionally, the presence of murine-derived *N*-glycolylneuraminic acid has been demonstrated on cetuximab, which is introduced by the manufacturing process [14]. Most or all humans make antibodies to this sialic acid; these antibodies have been shown to form immune complexes with cetuximab, but not panitumumab, in vitro [14].

The fully human nature of panitumumab was expected to decrease the rate of immunogenicity compared with therapeutic antibodies containing nonhuman coding sequences [15]. However, unique sequences in the complementarity determining regions (CDRs) and potential manufacturing-related modifications still provide the potential for panitumumab to be recognized as nonself by the human immune system, which could result in the development of anti-panitumumab antibody responses. As it is not known whether these immune responses could lead to clinically serious outcomes, the monitoring of patients who participated in clinical trials for the development of antibodies and an assessment of the impact of anti-panitumumab antibodies on pharmacokinetics and safety was a critical component of the panitumumab clinical development program.

The immunogenicity of panitumumab when administered as monotherapy was evaluated in clinical trials of patients with mCRC and other solid tumors. The incidence of binding antibodies to panitumumab (excluding predose and transient positive patients) was < 1% as detected by the acid dissociation enzyme-linked immunosorbent assay (ELISA) [16] and 4.6% as detected by the Biacore^® ^assay [1,16]. The incidence of neutralizing antibodies was 1.6%. The analysis described here examined the immunogenicity of panitumumab when administered as combination therapy with oxaliplatin- or irinotecan-based chemotherapy regimens in patients with refractory mCRC and explored the relationship between panitumumab immunogenicity and pharmacokinetic and safety profiles.

## Methods

### Patients and sample collection

Serum samples for anti-panitumumab antibody testing were collected from mCRC patients enrolled in 4 panitumumab clinical trials (20050184, 20060277, 20050181, and 20050203). Patients in these studies received irinotecan-based (including FOLFIRI) or oxaliplatin-based (FOLFOX) chemotherapies with or without panitumumab. Study 20050184 (STEPP) was a phase 2, open-label, randomized trial to compare pre-emptive skin toxicity therapy with reactive treatment in patients with mCRC receiving panitumumab plus irinotecan or FOLFIRI [17]. Study 20060277 (PRECEPT) was a phase 2, open-label, single-arm trial that estimated the effect of tumor *KRAS *status (wild-type or mutant) on efficacy endpoints in patients receiving panitumumab plus FOLFIRI as second-line therapy for mCRC [18]. Study 20050181 was a phase 3 randomized trial to evaluate panitumumab plus FOLFIRI combination therapy versus FOLFIRI alone as second-line therapy for mCRC [4]. Study 20050203 (PRIME) was a phase 3, randomized trial to evaluate the efficacy and safety of panitumumab in combination with FOLFOX4 versus FOLFOX4 alone as first-line therapy for mCRC [3]. Panitumumab was administered at 6.0 mg/kg every 2 weeks (Q2W) in all studies; patients in one arm of study 20050184 received panitumumab at 9.0 mg/kg every 3 weeks.

Per the study protocols, serum samples for immunogenicity assessments were collected from all panitumumab-treated patients prior to study treatment (baseline samples) and from all patients at the safety follow-up visit (postbaseline samples, obtained 30 ± 3 days after the administration of the last study treatment). Serum samples used for the measurement of panitumumab concentration (pharmacokinetic assessments) were collected from patients enrolled in study 20050181 before dosing on cycle 1 day 1 and cycle 2 day 1, every 8 weeks starting at week 8, and at the safety follow-up visit (30 ± 3 days after administration of the last dose).

*KRAS *mutation status was determined using DNA isolated from fixed tumor samples. Mutations in *KRAS *were detected using a *KRAS *mutation kit (DxS Ltd, Manchester, UK) as previously described [19]. *KRAS *status was available from 87 (95%) patients in study 20050184, 109 (95%) patients in study 20060277, 1083 (91%) patients in study 20050181, and 1096 (93%) patients in study 20050203.

Patients receiving both doses of panitumumab (6.0 mg/kg Q2W and 9.0 mg/kg Q3W) were included in the immunogenicity and safety analyses.

These studies were conducted in accordance with the principles for human experimentation as defined in the International Conference on Harmonization Good Clinical Practice guidelines and the principles of the Declaration of Helsinki. All studies were approved by the corresponding Investigational Review Boards, and informed consent was obtained from each patient after being advised of the potential risks and benefits as well as the investigational nature of the study.

### Anti-panitumumab antibody detection assays

**Figure 1:**
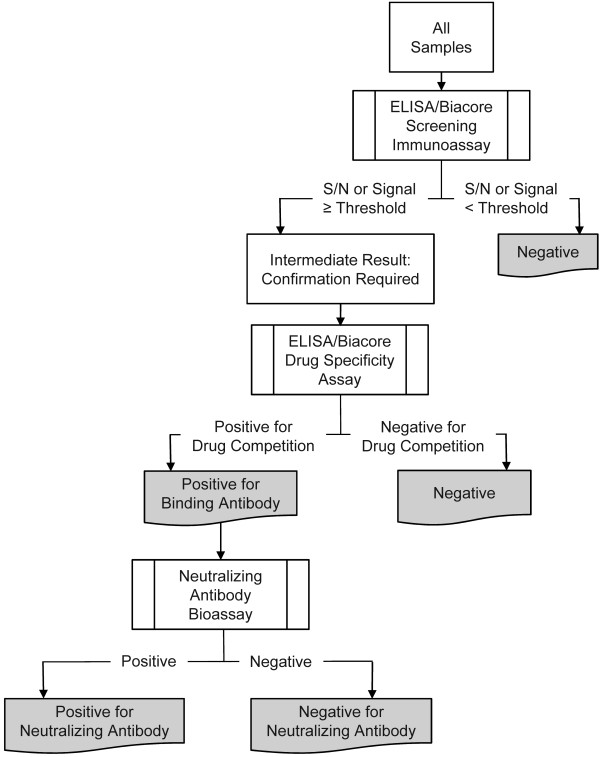
**Panitumumab immunogenicity testing strategy**. Three validated assays were used to detect the presence of anti-panitumumab antibodies. All clinical study samples were tested in two screening immunoassays (an acid-dissociation ELISA and a Biacore-based biosensor assay) to detect antibodies capable of binding to panitumumab. Samples that tested above the assay threshold and demonstrated reduction in response in the drug-competition specificity assay were reported as positive for binding antibodies and tested further in a cell-based neutralizing antibody bioassay. Assay thresholds were based on the upper bound of a one-sided 95% reference interval for the distribution of signals generated by serum samples from healthy subjects or cancer patients. S/N, signal-to-noise ratio.

Antibody samples were evaluated for the presence of anti-panitumumab antibodies according to the testing strategy depicted in Figure 1. The screening assays used to detect antibodies capable of binding to panitumumab, an ELISA and a Biacore assay, have been previously described [16]. All samples confirmed to be positive in either screening assay were further tested for antibodies capable of neutralizing the activity of panitumumab in vitro in a cell-based EGFR phosphorylation bioassay as previously described [16]. Immunogenicity assay characteristics are shown in Table 1. Developing antibodies were defined as antibodies that were observed only at a postbaseline time point(s) but not at the baseline time point. Pre-existing antibodies were defined as antibodies that were observed at the baseline time point.

**Table 1:** Immunogenicity assay characteristics

			Drug Tolerance^†^	
			
Assay	Assay Sensitivity*	LLRD	Anti-panitumumab	Panitumumab
ELISA	10 ng/mL	60 ng/mL	60 ng/mL 500 ng/mL	9 μg/mL 81 μg/mL
Biacore	1.8 μg/mL	1.8 μg/mL	4 μg/mL 20 μg/mL	1.9 μg/mL 7.8 μg/mL
Bioassay	~62.5 ng/mL	~125 ng/mL	~1 μg/mL	~2.5 μg/mL

*Intersection of the assay threshold with the rabbit polyclonal anti-panitumumab antibody dose-response curve.

**^†^**Level of anti-panitumumab antibodies that can be detected in the presence of the amount of panitumumab in the adjacent column.

LLRD, lower limit of reliable detection, the lowest concentration of the rabbit polyclonal anti-panitumumab antibody that could be reliably detected as positive when spiked into neat serum from multiple donors.

The assays and sample collection strategy were optimized to reduce drug interference with antibody detection. The performance of the immunogenicity assays was monitored by implementing a trending process utilizing Westgard multirules that tracked negative control and positive control values for each assay performed [20]. The observed low incidences of trending alarms and assay failures in the screening immunoassays and bioassay were indicative of assay stability.

### Serum panitumumab concentration immunoassay

Panitumumab concentrations in human serum were measured by a validated ELISA. Briefly, panitumumab was captured in microplate wells precoated with mouse anti-panitumumab antibody (Amgen Inc., Thousand Oaks, CA, USA). Horseradish peroxidase-labeled rabbit polyclonal anti-panitumumab antibody (Amgen Inc.) was added to the wells and allowed to react with the captured panitumumab. A colorimetric signal was produced after the addition of tetramethylbenzidine-peroxidase substrate solution. The optical density (OD) of the signal, measured at 450-650 nm, was proportional to the amount of captured panitumumab. The conversion of OD units to concentrations for the assay quality controls and samples was achieved through a computer software-mediated comparison to a standard curve on the same assay run, which was regressed according to a 5 PL (Auto Estimate) regression model with a weighting factor of 1/Y. The lower limit of quantitation was 400 ng/mL in human serum.

### Impact of immunogenicity on panitumumab pharmacokinetics

A population pharmacokinetic (PopPK) modeling and simulation approach was performed to evaluate the impact of immunogenicity on panitumumab pharmacokinetics by comparing the observed panitumumab concentrations of antibody-positive patients from study 20050181 with the predicted pharmacokinetic profiles based on PopPK parameters for antibody-negative patients.

A 2-compartment model with parallel linear and nonlinear (Michaelis-Menten) elimination pathways has been used to describe the disposition of panitumumab [21,22]. A previously developed and validated PopPK model [21] was updated by including the pharmacokinetic data from antibody-negative patients in study 20050181. The predictive check [23] was used to evaluate the validity of the updated PopPK model before subsequent analyses were performed. Using the updated PopPK parameters for antibody-negative patients, 1000 pharmacokinetic profiles were simulated for each antibody-positive patient according to the actual individual dosing history. The observed concentrations from each antibody-positive patient were superimposed with the model-predicted distribution. The pharmacokinetics of panitumumab in antibody-positive patients would be considered similar to antibody-negative patients if the proportions of the observed concentrations from antibody-positive patients falling within and outside the 90% prediction interval were not statistically different (chi-square test; α < 0.05) from hypothesized proportions, ie, 5% above, 5% below, and 90% within the interval. A sensitivity analysis was conducted to evaluate the minimum difference in panitumumab concentration between antibody-positive and antibody-negative patients that, given the available sample size, could be statistically significant.

### Impact of immunogenicity on panitumumab safety

The impact of immunogenicity on safety was evaluated through the review and assessment of the incidence of adverse events, potential infusion reactions, reasons for discontinuation from therapy, and total number of doses received while on study for panitumumab-treated antibody-positive patients and anti-panitumumab antibody-negative patients.

## Results

### Patients

A total of 1325 patients were tested for anti-panitumumab antibodies, including 558 patients who were treated with panitumumab plus oxaliplatin-based chemotherapy (study 20050203) and 767 patients treated with panitumumab plus irinotecan-based chemotherapy (studies 20050181, 20050184, and 20060277). Of these, 1225 patients (511 treated with panitumumab plus oxaliplatin and 714 treated with panitumumab plus irinotecan) had baseline samples available and 1124 patients (480 treated with panitumumab plus oxaliplatin and 664 treated with panitumumab plus irinotecan) had postbaseline samples available for testing. Over half of the patients were men and the population was predominantly white. Overall, 53% of the patients had tumors expressing wild-type *KRAS *(39% had mutant *KRAS *and 8% were unevaluable).

### Anti-panitumumab antibodies

The development of anti-panitumumab antibodies in patients receiving panitumumab in combination with chemotherapy (oxaliplatin- or irinotecan-based) occurred infrequently: 1.8% of patients developed binding antibodies and 0.2% of patients developed neutralizing antibodies (Table 2). The incidence of developing antibodies was low in patients with tumors expressing wild-type and mutant *KRAS *(2.0% and 1.4% binding antibodies and 0.2% and 0.2% neutralizing antibodies, respectively). The incidence of developing antibodies was low in patients treated with oxaliplatin-based chemotherapy (2.9% binding antibodies and 0.4% neutralizing antibodies) and in patients treated with irinotecan-based chemotherapy (0.9% binding antibodies and 0% neutralizing antibodies). Pre-existing binding and neutralizing antibodies were detected in 3.8% and 0.4% of all patients before the start of any investigational product, respectively.

**Table 2:** Incidence of anti-panitumumab antibodies

	Wild-Type *KRAS*		Mutant *KRAS*		All Patients*	
	
	Either Biacore or ELISA	Bioassay	Either Biacore or ELISA	Bioassay	Either Biacore or ELISA	Bioassay
**Total Antibody Incidence, n_1_/n_2 _(%)**						
20050184	6/48 (12.5)	1/48 (2.1)	0/37 (0)	0/37 (0)	6/93 (6.5)	1/93 (1.1)
20060277	0/64 (0)	0/64 (0)	1/45 (2.2)	1/45 (2.2)	1/115 (0.9)	1/115 (0.9)
20050181	11/288 (3.8)	0/288 (0)	9/223 (4.0)	1/223 (0.4)	22/559 (3.9)	1/559 (0.2)
20050203 (Pmab + OX)	21/308 (6.8)	3/308 (1.0)	10/206 (4.9)	1/206 (0.5)	36/558 (6.5)	4/558 (0.7)
Pmab + IRI	17/400 (4.3)	1/400 (0.3)	10/305 (3.3)	2/305 (3.3)	29/767 (3.8)	3/767 (0.4)
Pmab + IRI or OX	38/708 (5.4)	4/708 (0.6)	20/511 (3.9)	3/511 (0.6)	65/1325 (4.9)	7/1325 (0.5)
**Pre-existing Antibody Incidence, n_3_/n_4 _(%)**						
20050184	4/48 (8.3)	1/48 (2.1)	0/37 (0)	0/37 (0)	4/93 (4.3)	1/93 (1.1)
20060277	0/64 (0)	0/64 (0)	1/45 (2.2)	1/45 (2.2)	1/115 (0.9)	1/115 (0.9)
20050181	11/252 (4.4)	0/252 (0)	7/210 (3.3)	1/210 (0.5)	19/506 (3.8)	1/506 (0.2)
20050203 (Pmab + OX)	11/282 (3.9)	2/282 (0.7)	7/188 (3.7)	0/188 (0)	22/511 (4.3)	2/511 (0.4)
Pmab + IRI	15/364 (4.1)	1/364 (0.3)	8/292 (2.7)	2/292 (0.7)	24/714 (3.4)	3/714 (0.4)
Pmab + IRI or OX	26/646 (4.0)	3/646 (0.5)	15/480 (3.1)	2/480 (0.4)	46/1225 (3.8)	5/1225 (0.4)
**Developing Antibody Incidence, n_5_/n_6 _(%)**						
20050184	2/37 (5.4)	0/37 (0)	0/26 (0)	0/26 (0)	2/68 (2.9)	0/68 (0)
20060277	0/42 (0)	0/42 (0)	0/30 (0)	0/30 (0)	0/75 (0)	0/75 (0)
20050181	0/255 (0)	0/255 (0)	3/205 (1.5)	0/205 (0)	4/501 (0.8)	0/501 (0)
20050203 (Pmab + OX)	10/264 (3.8)	1/264 (0.4)	3/178 (1.7)	1/178 (0.6)	14/480 (2.9)	2/480 (0.4)
Pmab + IRI	2/334 (0.6)	0/334 (0)	3/261 (1.1)	0/261 (0)	6/644 (0.9)	0/644 (0)
Pmab + IRI or OX	12/598 (2.0)	1/598 (0.2)	6/439 (1.4)	1/439 (0.2)	20/1124 (1.8)	2/1124 (0.2)

*All Patients includes patients with wild-type, mutant, and unknown *KRAS *status.

Pmab, panitumumab; IRI, irinotecan-based chemotherapy; OX, oxaliplatin-based chemotherapy; n_1_, number of patients with a positive antibody result at any time point; n_2_, number of patients with at least one immunoassay result; n_3_, number of patients with a positive antibody result at or before baseline; n_4_, number of patients with an immunoassay antibody result at or before baseline; n_5_, number of patients with negative or no antibody result at or before baseline and a positive antibody result at a postbaseline time point; n_6_, number of patients with at least one postbaseline immunoassay antibody result

### Impact of immunogenicity on panitumumab pharmacokinetics

Of the 22 patients with pre-existing and post-treatment developing anti-panitumumab antibodies in study 20050181, 38 samples from 11 patients were available for evaluation of panitumumab concentration. In addition, 68 samples from 53 antibody-negative patients in study 20050181 were analyzed for panitumumab concentration. Because of the low rate of immunogenicity in study 20050181 and limited availability of pharmacokinetic samples, the concentration data from patients with pre-existing and post-treatment developing antibody responses were combined for analysis.

Among 38 observed concentrations from the antibody-positive patients, 2 (5%) were below, 5 (13%) were above, and 31 (82%) were within the 90% prediction interval derived from pharmacokinetic profiles of antibody-negative patients. The proportions were not statistically different (*P *= 0.0685) from the hypothesized proportions (Table 3) and were similar to those for the antibody-negative patients (*P *= 0.8807), suggesting that no marked difference in the observed panitumumab concentrations was observed between the antibody-positive and antibody-negative patients.

**Table 3:** Distribution of the observed concentrations of panitumumab relative to the 90% predictive interval

Distribution in Relation to 90% Prediction Interval	Antibody-Positive Patients				Antibody-Negative Patients				*P*-value^†^
			
	N	%	95% CI	*P*-value*	N	%	95% CI	*P*-value*	
Below	2	5.3	0.6 -17.8	0.07	3	4.4	0.9 - 12.4	0.13	0.88
Above	5	13.2	4.4 - 28.1		7	10.3	4.2 - 20.1		
Within	31	81.6	65.7 - 92.3		58	85.3	74.6 - 92.7		

*Chi-square test with hypothesized proportions (5% below, 5% above, 90% within).

**^†^**Chi-square test of antibody-positive proportion vs antibody-negative proportion.

**Figure 2:**
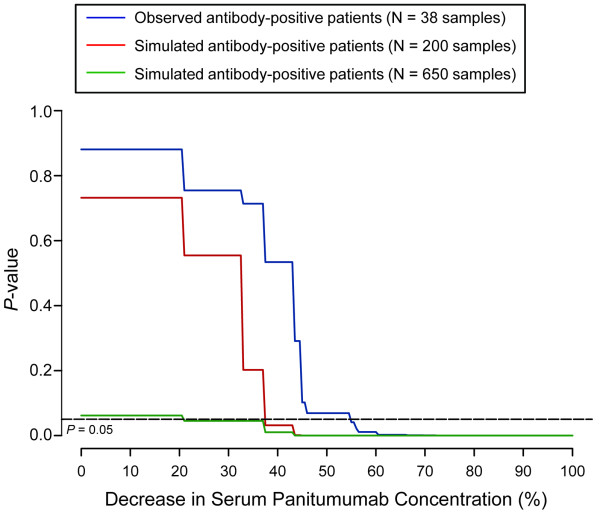
**Sensitivity analysis**. The sensitivity analysis estimated the minimum difference in panitumumab concentration between antibody-positive and antibody-negative samples that could be statistically significant (*P *< 0.05) with respect to the model prediction. Results show that the current observed sample size for pharmacokinetic testing (n = 38) from antibody-positive patients was only adequate to detect a difference of > 55%. Approximately 200 and 650 samples from antibody-positive patients would be required to detect differences of 38% and 20%, respectively.

Results of the sensitivity analysis showed that a statistically significant effect of immunogenicity would have been observed if the serum concentrations of antibody-positive patients were at least 55% lower than the current observed values given the current sample size. To further evaluate the impact of the sample size on the analysis, additional analyses were performed and the results showed that 200 and 650 samples would be needed to detect a 38% and 20% difference in panitumumab serum concentration, respectively (Figure 2).

### Impact of immunogenicity on panitumumab safety profiles

No apparent difference in the rate of grade 3 and grade 4 adverse events (as graded per National Cancer Institute Common Terminology Criteria for Adverse Events [NCI CTCAE] version 3.0) was observed between patients who tested positive for anti-panitumumab antibodies and those who tested negative (Table 4). Although the incidence of grade 5 events was higher in patients testing positive for developing antibodies (n = 4; 19%) than in antibody-negative patients (n = 82; 6%), following a medical review of these cases, all were reported in the setting of progression of the underlying disease. The number of chemotherapy cycles received varied among patients; no apparent relationship between antibody status and number of cycles received was observed. Three patients experienced infusion reactions deemed related to panitumumab. Two patients (1 predose-positive and 1 developing antibody-positive) experienced grade 1 (mild) infusion reactions reported as vomiting and fever. One patient (predose-positive) had a grade 2 (moderate) infusion reaction of hypersensitivity, which was reported on study day 272 following multiple administrations of panitumumab. The event of hypersensitivity was confounded by the coadministration of oxaliplatin. Although the analysis of the impact of immunogenicity on safety was limited by the small number of antibody-positive patients, there was no evidence of an altered safety profile found in patients who tested positive for pre-existing or post-treatment developing anti-panitumumab antibodies compared to the safety profile of those patients who tested negative.

**Table 4:** Summary of adverse events (safety analysis set*)

	Antibody Negative (N = 1317)	Antibody Positive (N = 65)	Predose Positive (N = 46)	Developing Antibody Positive (N = 21)
Patients with any adverse event**^†^**, n (%)	1312 (100)	65 (100)	46 (100)	21 (100)
Worst grade of 3**^‡^**	716 (54)	26 (40)	18 (39)	8 (38)
Worst grade of 4**^‡^**	264 (20)	18 (28)	15 (33)	4 (19)
Worst grade of 5**^‡^**	82 (6)	6 (9)	2 (4)	4 (19)
Any serious adverse event	544 (41)	32 (49)	23 (50)	10 (48)
Patients with any adverse event leading to permanent discontinuation of any study drug, n (%)	299 (23)	16 (25)	8 (17)	8 (38)
Not serious	217 (16)	12 (18)	6 (13)	6 (29)
Serious	108 (8)	6 (9)	3 (7)	3 (14)
Patients with any treatment-related adverse event^§^, n (%)	1298 (99)	65 (100)	46 (100)	21 (100)
Worst grade of 3**^‡^**	739 (56)	30 (46)	20 (43)	11 (52)
Worst grade of 4**^‡^**	204 (15)	15 (23)	13 (28)	2 (10)
Worst grade of 5**^‡^**	13 (1)	1 (2)	0	1 (5)
Any serious adverse event	314 (24)	20 (31)	14 (30)	6 (29)
Patients with any adverse event leading to permanent discontinuation of any study drug, n (%)	251 (19)	11 (17)	5 (11)	6 (29)
Not serious	200 (15)	9 (14)	4 (9)	5 (24)
Serious	64 (5)	2 (3)	1 (2)	1 (5)

*The safety analysis set included all patients who were enrolled, randomized, and received at least one dose of study treatment in studies 20050203, 20050181, 20050184, and 20060277. Patients not testing positive by ELISA, Biacore, and bioassay or with all antibody results missing were included.

**^†^**Adverse events were coded using the Medical Dictionary for Regulatory Activities (MedDRA) version 12.0.

**^‡^**Severity was graded using the Common Terminology Criteria for Adverse Events (CTCAE) version 3.0, with the exception of some dermatology/skin adverse events, which were graded using CTCAE version 3.0 with modifications. Fatal adverse events were classified as grade 5.

^§^The investigator considered there to be a reasonable possibility that the event may have been caused by study drug.

## Discussion

The panitumumab immunogenicity testing strategy utilized two validated screening immunoassays to detect the presence of all antibodies capable of binding to panitumumab. The screening immunoassays were chosen to provide the optimal combination of sensitivity and drug tolerance (ELISA) and the ability to detect antibodies of various affinities (Biacore) [16]. A cell-based bioassay was used to detect antibodies with neutralizing activity. Despite the use of these validated and sensitive assays, the development of anti-panitumumab antibodies in combination chemotherapy patients was detected infrequently. Developing antibody incidences did not appear to be affected by tumor *KRAS *status (wild-type or mutant) or combination chemotherapy regimen (oxaliplatin- or irinotecan-based).

Pre-existing binding and neutralizing antibodies were detected prior to the administration of panitumumab in 3.8% and 0.4% of patients, respectively. Positive results from these baseline samples may be due to the presence of cross-reacting antibodies generated against antigens that share a similar epitope with panitumumab. The presence of pre-existing antibodies in panitumumab-treated patients did not appear to affect the postdose antibody state of these patients. In addition, patients who tested positive for pre-existing antibodies did not appear to have altered safety profiles.

The broad, nonspecific cytotoxic effects of chemotherapy have the potential to impair the immune system, which could reduce the incidence of anti-panitumumab antibodies in patients receiving panitumumab in combination with chemotherapy. Both oxaliplatin and irinotecan have gastrointestinal toxicities [24,25], and have the potential to affect local and systemic immunity. The effects of chemotherapy on acquired immunity may have affected the development of anti-panitumumab antibodies in patients receiving chemotherapy plus panitumumab. The incidence of binding antibodies in the monotherapy setting (excluding predose and transient positive patients) was 3/613 (< 1%) as detected by the acid dissociation ELISA and 28/613 (4.6%) as detected by the Biacore assay [1], and neutralizing antibodies were detected in 10/613 (1.6%) of the patients [1]. The incidence of developing binding and neutralizing antibodies in the combination chemotherapy setting described here was similar but slightly lower (1.0% as detected by the ELISA, 0.8% as detected by the Biacore assay and 0.2% as detected by the neutralizing assay).

Based on available pharmacokinetic data from study 20050181, the phase 3 study of panitumumab plus FOLFIRI for second-line treatment of mCRC, no evidence of an altered panitumumab pharmacokinetic profile was found in patients who received panitumumab 6.0 mg/kg Q2W and tested positive for pre-existing or developing anti-panitumumab antibodies. The results from study 20050181 were in agreement with previously published observations that there were no apparent differences in steady-state AUC, C_max_, and C_min _between patients who developed anti-panitumumab antibodies and those who did not [21,22]. A sensitivity analysis was conducted to show that even though the pharmacokinetic data were limited, a statistically significant effect of immunogenicity would have been observed if the difference in serum concentrations between antibody-positive and antibody-negative patients was greater than 55%. Therefore, this analysis ruled out the possibility that immunogenicity would cause a > 55% decrease in the panitumumab serum concentrations. Since the relationship between pharmacokinetics and efficacy has not been established, it is unclear what level of decrease in panitumumab serum concentration would result in a change in efficacy. By assuming a < 20% difference would be biologically unimportant, additional simulations were conducted to understand the sample size required to detect that level of difference. The result suggested that approximately 650 samples from antibody-positive patients would be required to detect a 20% difference in pharmacokinetics. To obtain this larger number of samples, it would require either fewer samples (ie, sparse sampling) from a larger antibody-positive population or more samples (ie, intensive sampling) from a smaller antibody-positive population, both of which would be challenging, considering the low rate of immunogenicity for panitumumab.

Overall, there did not appear to be an association between the development of antibodies and safety outcomes. Higher incidences of adverse events observed in patients who were antibody negative at baseline and developed antibodies during the conduct of the study (n = 21) compared with those who were antibody negative throughout the study (n = 1317) may be related to the small sample size. Grade 5 events in antibody-positive patients occurred in the setting of disease progression, and serious adverse events were similar in type and nature to those reported in patients who did not develop anti-panitumumab antibodies.

Although panitumumab was expected to have a low rate of immunogenicity compared to therapeutic antibodies containing nonhuman coding sequences, unique sequences in the CDRs could still be immunogenic. An in silico analysis to evaluate the potential risk of panitumumab sequence-associated immunogenicity suggested that panitumumab light and heavy chains do not contain any non-tolerant agretopes predicted to be able to bind to the eight most common HLA-DRB1 alleles. The low risk of panitumumab immunogenicity was confirmed by the results of antibody testing in combination chemotherapy clinical trials, which indicated that patients treated with panitumumab in combination with irinotecan- or oxaliplatin-based chemotherapy infrequently developed antibodies against panitumumab.

## Conclusions

In summary, the immunogenicity of panitumumab in the combination chemotherapy setting was infrequent and was similar to the immunogenicity observed in the monotherapy setting. This low rate of immunogenicity may be attributed to the fully human nature of panitumumab. Additionally, the presence of anti-panitumumab antibodies did not appear to alter pharmacokinetic or safety profiles of panitumumab.

## Competing interests

All authors are employees and shareholders of Amgen Inc.

## Authors' contributions

DW and JJP developed the methods and coordinated the implementation of binding and neutralizing antibody testing. AC performed the PopPK analysis and interpreted results. CYW, JJPR, and BBY made substantial contributions to the PopPK analysis planning and interpreted the results. KZ performed the statistical analysis of the antibody data. JW performed the analysis of the impact of immunogenicity on safety. AK and SG supervised development and implementation of the neutralizing antibody testing method. SJS contributed to the interpretation of antibody data. NC and MS developed and implemented the immunogenicity testing strategy and made substantial contributions to data interpretation. All authors contributed to the writing of the manuscript or critically reviewing the manuscript for technical and intellectual content. All authors approved the final draft of the manuscript.

## Pre-publication history

The pre-publication history for this paper can be accessed here:

http://www.biomedcentral.com/1472-6904/11/17/prepub
